# Subjective time compression induced by continuous action

**DOI:** 10.1038/s41598-021-92946-x

**Published:** 2021-06-29

**Authors:** Sayako Ueda, Shingo Shimoda

**Affiliations:** RIKEN Center for Brain Science, TOYOTA Collaboration Center, RIKEN, 2271-130 Anagahora, Shimoshidami, Moriyama-ku, Nagoya, Aichi 463-0003 Japan

**Keywords:** Psychology, Human behaviour

## Abstract

Increasing evidence indicates that voluntary actions can modulate the subjective time experience of its outcomes to optimize dynamic interaction with the external environment. In the present study, using a temporal reproduction task where participants reproduced the duration of an auditory stimulus to which they were previously exposed by performing different types of voluntary action, we examined how the subjective time experience of action outcomes changed with voluntary action types. Two experiments revealed that the subjective time experience of action outcomes was compressed, compared with physical time, if the action was performed continuously (Experiment 1), possibly enhancing the experience of controlling the action outcome, or if the action was added an extra task-unrelated continuous action (Experiment 2), possibly reflecting different underlying mechanisms from subjective time compression induced by the task-related continuous action. The majority of prior studies have focused on the subjective time experience of action outcomes when actions were performed voluntarily or not, and no previous study has examined the effects of differences in voluntary action types on the subjective time experience of action outcomes. These findings may be useful in situations in which people wish to intentionally compress their own time experience of daily events through their voluntary actions.

## Introduction

The sense of time, despite being a fundamental sensory ability in daily life, lacks a specific sensory organ, in contrast with other basic sensory abilities, such as vision and audition. Instead, the sense of time is thought to be encoded by the motor system^1^. Recent meta-analyses^2–7^ have demonstrated the strong involvement of the supplementary motor area (SMA) in time processing, which has traditionally been considered to contribute to the voluntary control of movement. Strong evidence has been provided by studies of patients with movement and motor system disorders such as Parkinson’s disease (PD) and tremor, which are characterized by dysfunctions in time processing^8–11^. Pastor et al.^8^ demonstrated that patients with PD underestimated the duration of a time interval in a verbal time estimation task, and exhibited overproduction of time intervals when required to reproduce a short time sample. These findings suggest that the sense of time shares a common brain system with motor control, even though these two functions are intuitively independent.

Increasing evidence indicates that voluntary actions can modulate the subjective time experience of its outcomes, regardless of the sensory modality, in order to optimize dynamic interactions with the external environment through voluntary action^12–20^. Haggard et al.^12^ demonstrated that the interval of time between a single voluntary action (a keypress) and auditory feedback (a tone) was perceived to be shorter compared with cases in which an involuntary transcranial magnetic stimulation (TMS)-induced action initiated the tone. Imaizumi et al.^16^. found that the duration of a visual event which was initiated and ended by the participant’s voluntary keypresses was subjectively estimated as shorter compared with cases in which the experimenter moved the participant’s finger so that keypresses were executed involuntarily. These findings suggest that voluntary actions can subjectively compress the temporal interval between a voluntary action and its outcomes, and the temporal duration of action outcomes. Such subjective time compression is considered to play a role in maintaining the experience of controlling action outcomes in the external environment. In addition, the temporal duration of action outcomes can also be dilated. Using a task in which participants voluntarily performed a hand action, such as making a fist while watching video feedback of their hand action and judging the perceived duration of the video feedback, Imaizumi et al.^20^ found that the duration was perceived as longer when participants viewed video feedback of their own hand action compared with a pre-recorded video of another person’s hand action. Yarrow et al.^19^ demonstrated that a reaching action resulted in overestimation of the duration of a consequent vibrotactile event. Such subjective time dilation has been considered to play a role in maintaining the consistency of the perceptual experience of action outcomes in the external environment.

All of these previous studies^12–20^ have focused on the subjective time experience of action outcomes when actions are performed voluntarily or not. However, in daily life, various types of voluntary actions can be performed at different levels of goal setting. For example, a goal such as grinding coffee beans can be accomplished by waiting after pressing a button on an electric coffee grinder, consisting of a single voluntary action, but also by turning the handle of a manual coffee grinder, constituting continuous voluntary action. In this case, the subjective time experience of grinding the coffee beans (i.e., the action outcome) accomplished by a continuous voluntary action may differ from that accomplished by a single action. In addition, some voluntary actions can be performed continuously without a specific goal, such as knee jiggling while another main action task (e.g., a keyboard typing task) is performed. Such task-unrelated continuous voluntary action may influence the subjective time experience of outcomes of the main action. However, no previous studies have focused on the effect of differences in voluntary action types on the subjective time experience of action outcomes. Elucidating this issue may have valuable applications.

In the current study, we investigated the effects of differences in voluntary action types on the subjective time experience of action outcomes, focusing on the continuity of action. To achieve this goal, we used a temporal reproduction task, in which participants reproduced the duration of an auditory stimulus (a tone) to which they were previously exposed by performing different types of voluntary actions involving common auditory feedback consisting of a different tone from the stimulus tone. In Experiment 1, we contrasted two different voluntary action types with a common goal to investigate whether the subjective time experience of a feedback tone (i.e., the action outcome) while reproducing durations is compressed, dilated, or unaffected by two different action types: a combination of single actions, where the feedback tone was started by a keypress then ended by another keypress, and a continuous action, where the tone was produced by continuously turning a steering wheel. Based on previous studies^12–20^, if subjective time compression is induced by the action, the action plays a role in maintaining the experience of controlling the feedback tone, whereas, if subjective time dilation is induced by the action, it could be concluded that the action plays a role in maintaining the consistency of the perceptual experience of the feedback tone. In Experiment 2, we contrasted two common continuous voluntary actions (continuously turning a wheel), each with a different goal; a task-related continuous action and a task-unrelated continuous action, where participants reproduced the duration by performing the continuous action (continuously turning a wheel) producing the feedback tone, or by performing the combination of single actions (two keypresses) starting/ending the feedback tone as they kept performing an extra continuous action (continuously turning a wheel) at the same time (i.e., the extra continuous wheel turning had nothing to do with the feedback tone). This comparison allowed us to determine whether the pattern of results in the continuous action condition found in Experiment 1 was specific to situations in which the continuous action was performed in task-related manner.

## Methods

### Participants

Twenty-six naïve adults (16 females; 10 males) aged 20 to 39 years (M = 28.3 years; SD = 6.5 years) participated in this study (13 adults per experiment) and received monetary compensation for their participation. The sample size was determined with a power analysis using G*Power^21^ using the solution proposed by David Morse^22,23^ with parameters which was decided based on a preliminary study. The analysis indicated that for the interaction of a two-way within-subjects repeated measures analysis of variance (ANOVA), a minimum sample size of 13 participants was needed to detect a medium effect (f = 0.25) with 80% power and an alpha level of 0.05. All participants had normal or corrected-to-normal vision. All participants were right-handed according to the Edinburgh Handedness Inventory^24^. In addition, participants were ascertained to have normal visuomotor functions, as assessed by the Grooved pegboard test (Lafayette Instruments, Lafayette, IN). Written informed consent was obtained in accordance with a protocol approved by the RIKEN Research Ethics Committee [Wako3 30-13(2)]. All methods were carried out in accordance with relevant guidelines and regulations.

### Apparatus, setup and procedure

**Figure 1:**
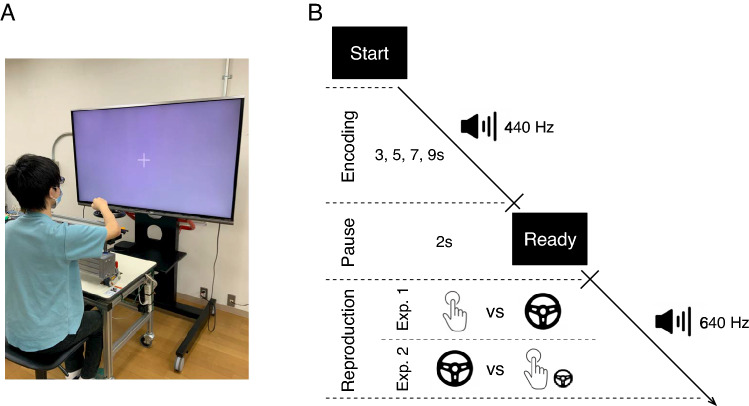
Apparatus and the temporal reproduction task. Participants were seated in front of a table with a keypad and a steering wheel which were placed in front of a monitor (**A**). In the temporal reproduction task (**B**), participants reproduced the duration of previously presented tones by pressing the key twice (the single action condition) or turning the steering wheel (the continuous action condition) or pressing the key twice while turning the steering wheel (the extra continuous action condition).

The apparatus and task design are shown in Fig. 1. The experiments were conducted in a dimly lit room. Participants were seated at a distance of 60 cm from a 60-inch LCD monitor (LC60XL10, SHARP Corp., Japan). A keypad (BSTK10, BUFFALO Inc., Japan) and a steering wheel (diameter of 20 cm, see also Itkonen et al.^25^) were placed on a table in front of them (Fig. 1A : We have received informed consent for publication from the volunteer to have the face of the volunteer appear in the figure.). In the temporal reproduction task, participants were instructed to reproduce the duration of previously presented tones (with durations of 3, 5, 7 and 9 s) by pressing the key or turning the steering wheel. Each trial began with a “Start” prompt presented on the monitor. Immediately after pressing the key with the right hand, the fixation cross was presented, and an encoding phase was started, in which a 440 Hz tone was presented for one of four durations (the encoding tone). After the tone, the fixation cross immediately disappeared and a “Ready” prompt was presented for 2 s, followed by a reproduction phase, in which the fixation cross was presented again and a 640 Hz tone (the reproduction tone) was created (Fig. 1B). Two different tones for the encoding and reproduction phases were used here to ensure that participants could not potentially use a sound feature of the encoding tone as a cue to reproduce the encoded duration in the reproduction phase. In Experiment 1, after the reproduction phase had started, participants were either instructed to start the reproduction tone by pressing the key, and to end it by pressing the key with the right hand when they estimated that the duration of the encoding tone had elapsed (single action condition), or, they were instructed to keep creating the reproduction tone by continuously turning the steering wheel outward with the right hand until they estimated that the duration of the encoding tone had elapsed (continuous action condition). In Experiment 2, one condition was the same as the continuous action condition while the other condition was similar to the single action condition, except that participants were instructed to continuously turn the steering wheel outward with the left hand during the reproduction tone (extra continuous action condition). The reason the left hand was used to perform the extra (i.e., task-unrelated) continuous action in Experiment 2 was to ensure that the task-related actions (continuously turning the steering wheel in the continuous action condition and two keypresses in the extra continuous action condition) were performed using the right hand. Participants were additionally instructed not to use methods such as counting or tapping to complete the task and to turn the steering wheel at a constant speed which was predetermined to be comfortable for participants. The steering wheel position was recorded at intervals of 1/60 s. Presentation of stimuli and recording of the steering wheel’s position were controlled by a computer, using MATLAB software with the Psychtoolbox extension^26^. In each experiment, half of the participants started with one task condition, and the other half started with the other task condition, to counter-balance the order of condition (single action vs. continuous action in Experiment 1, continuous action vs. extra continuous action in Experiment 2) across participants. To familiarize themselves with the task, participants received five practice trials with an encoding duration of 2 s before starting each task condition. Following the practice session, for each task condition, each participant completed 24 trials (experimental session), comprising six trials involving each duration (i.e., 3, 5, 7 and 9 s), in a random order. Participants took 1-min breaks between the practice and experimental sessions. The experiment lasted for 25 min on average.

### Data analysis

For each trial, temporal reproduction performance was evaluated using the temporal reproduction error, which was calculated by normalizing (i.e., dividing) the reproduced duration by the encoded duration^27–29^. A value of 1 indicated that the encoded duration was accurately reproduced, a value > 1 indicated that the reproduced encoded duration was prolonged (i.e., over-reproduction), and a value < 1 indicated that the reproduced encoded duration was short (i.e., under-reproduction). Within the dominant theoretical framework of temporal processing: the internal clock model^30–33^, which includes a pacemaker that produces ticks that are counted by an accumulator, over-reproduction can be explained by the slowed pace of ticks of an internal clock, while under-reproduction can be explained by the quickened pace of ticks of the internal clock. When the pace of ticks slows, the duration is expected to be subjectively perceived as shorter, due to the accumulation of fewer pacing ticks (i.e., subjective time compression). Conversely, when the pace of ticks quickens, the duration is expected to be subjectively perceived as longer, due to the accumulation of more pacing ticks (i.e., subjective time dilation). Therefore, we used a value > 1 as an index of subjective time compression and a value < 1 as an index of subjective time dilation for the reproduction tone. In addition, the performance of continuous action was evaluated using the speed of action, which was calculated as the mean steering wheel velocity while turning the steering wheel in the reproduction phase for each trial in the continuous and extra continuous action task conditions. This method was used to examine the relationship with temporal reproduction performance.

Temporal reproduction error was submitted to a one-sample t-test to compare the values with 1, to determine whether the reproduced duration was compressed, dilated or perfectly accurate, then submitted to a two-way repeated measures ANOVA with task condition (i.e., for Experiment 1, single and continuous action conditions; for Experiment 2, continuous and extra continuous action conditions) and duration condition (i.e., 3, 5, 7 and 9 s) as repeated measures to compare between task conditions. When a significant interaction between task and duration condition was found, subsequent simple main effect analyses were performed. When a significant simple main effect of duration condition was found, multiple comparisons were performed, and *p*-values were corrected using Shaffer’s modified sequentially rejective Bonferroni procedure^34^. When the interaction was not significant but a significant main effect of duration condition was found, subsequent multiple comparisons using Shaffer’s modified sequentially rejective Bonferroni procedure^34^ were performed. In addition, to examine the relationship between the temporal reproduction error and the mean steering wheel velocity, for each duration condition, we calculated Pearson’s correlation coefficients, then conducted a one-sample t-test to compare the mean correlation coefficients with a value of 0. The significance threshold was set at *p* < 0.05 for all above tests. Statistical analysis was conducted using R software (version 3.3.2. for Mac, R). In addition, to support the above tests, we conducted Bayes factor (BF) analyses to calculate and additionally report BF values using JASP software version 0.14.1 (JASP Team; https://jasp-stats.org/) with JASP’s default prior scales (for performing Bayesian multiple comparisons, the posterior odds were corrected^35^). BF values indicate the extent to which obtained data support an alternative hypothesis over a null hypothesis. The value of BFs ranges from 0 to infinity. A BF value from 1 to 3 indicates weak, a BF value from 3 to 10 indicates moderate and a BF value more than 10 indicates strong support for the alternative hypothesis. By contrast, a BF value from 1 to 0.33 indicates weak, a BF value from 0.33 to 0.1 indicates moderate and a BF value less than 0.1 indicates strong support for the null hypothesis^36,37^. In the present context, the alternative hypothesis for a Bayesian one-sample t-test to compare temporal reproduction error with 1 was that the values were different from 1. The alternative hypothesis for a Bayesian two-way repeated measures ANOVA with task condition and duration condition to compare between task conditions was that the values were different between task conditions. The alternative hypothesis for a Bayesian one-sample t-test to compare the mean correlation coefficients with 0 was that the values were different from 0.

### Ethics approval and consent to participate

All experiments were conducted in accordance with a protocol approved by the RIKEN Research Ethics Committee (Wako3 30-13(2)).

## Results

### Experiment 1

**Figure 2:**
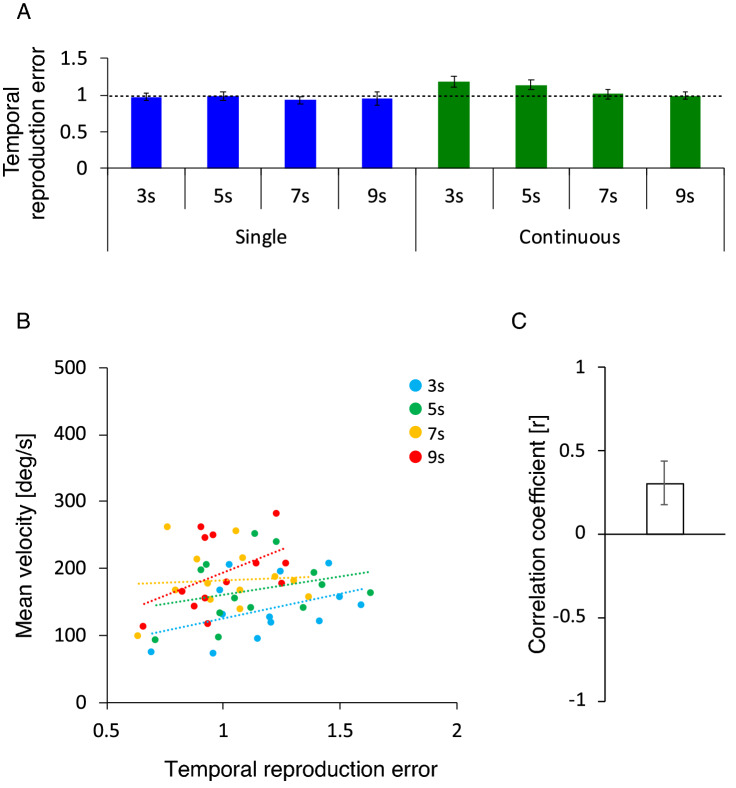
Average temporal reproduction error for the single and continuous action conditions (**A**), regression lines fitted to each duration condition’s temporal reproduction error and steering wheel velocity for the continuous action condition (**B**), and its average correlation coefficient (**C**). Statistical comparisons revealed that, compared with 1 (a dotted line), the temporal reproduction errors in the continuous action condition were higher in the 3-s and 5-s conditions, but identical in the 7-s and 9-s conditions, and the temporal reproduction errors in the single action condition were identical in the all duration conditions (**A**). The slopes of the regression lines appeared to be positive (**B**) and the average correlation coefficient was significantly higher than 0 (**C**). Error bars represent 95% confidence intervals.

Figure 2A shows the average temporal reproduction error for the single and continuous action conditions. The temporal reproduction errors in the single action condition were lower than 1 (i.e., perfectly accurate reproduction), while those in the continuous action condition were higher than 1, particularly in the 3-s, 5-s and 7-s conditions. A one-sample t-test showed that, compared with a value of 1, temporal reproduction errors in the single action condition were not significantly different in all duration conditions (3 s: *t*[12]  = 0.41, *p* = 0.69, *d* = 0.11, *BF* = 0.30, 5 s: *t*[12] = 0.33, *p* = 0.75, *d* = 0.09, *BF* = 0.29, 7 s: *t*[12] = 1.59, *p* = 0.18, *d* = 0.44, *BF* = 0.76, 9 s: *t*[12] = 0.93, *p* = 0.37, *d* = 0.26, *BF* = 0.40). In contrast, the temporal reproduction errors in the continuous action condition were significantly higher in the 3-s condition (*t*[12] = 2.65, *p* = 0.02, *d* = 0.73, *BF* = 3.15), marginally higher in the 5-s condition (*t*[12] = 2.05, *p* = 0.06, *d* = 0.57, *BF* = 1.35), but not significantly different in the 7-s and 9-s conditions (7 s: *t*[12] = 0.23, *p* = 0.81, *d* = 0.06, *BF* = 0.29, 9 s: *t*[12] = 0.09, *p* = 0.92, *d* = 0.03, *BF* = 0.28). Besides, both task conditions in Fig. 2A showed a similar trend (i.e., the temporal reproduction errors decreased with increasing duration lengths). This trend appeared to be particularly strong in the continuous action condition. A two-way repeated measures ANOVA showed the expected main effects of task condition (*F*[1, 12] = 7.30, *p* = 0.019, *ηp*^2^ = 0.38, *BF* = 350.39) and the duration condition (*F*[3, 36] = 4.77, *p* = 0.007, *ηp*^2^ = 0.28, *BF* = 4.22), and a significant interaction between task and duration condition (*F*[3, 36] = 2.92, *p* = 0.047, *ηp*^2^ = 0.20, *BF* = 1.85). We then performed subsequent simple main effect analyses and found expected significant simple main effects of task condition (i.e., Continuous > Single) in the 3-s and 5-s conditions (*F*[1, 12] = 15.00, *p* = 0.002, *ηp*^2^ = 0.56, *BF* = 17.94, *F*[1, 12] = 9.33, *p* = 0.01, *ηp*^2^ = 0.44, *BF* = 5.35, respectively) but not in the 7-s and 9-s conditions (*F*[1, 12] = 1.52, *p* = 0.24, *ηp*^2^ = 0.11, *BF* = 0.73, *F*[1, 12] = 0.39, *p* = 0.55, *ηp*^2^ = 0.03, *BF* = 0.43, respectively), revealing an expected significant simple main effect of duration condition in the continuous but not the single action condition (*F*[3, 36] = 6.77, *p* = 0.001, *ηp*^2^ = 0.36, *BF* = 33.73, *F*[3, 36] = 0.51, *p* = 0.68, *ηp*^2^ = 0.04, *BF* = 0.17, respectively). Post-hoc tests for the significant simple main effect of the duration condition in the continuous action condition revealed that the temporal reproduction error was significantly larger in the 3-s condition compared with the 7-s and 9-s conditions (*t*[12] = 3.09, *p* = 0.029, *d* = 0.85, *BF* = 6.00, *t*[12] = 4.26, *p* = 0.007, *d* = 1.18, *BF* = 36.47, respectively), marginally larger in the 5-s compared with the 9-s condition (*t*[12] = 2.65, *p* = 0.06, *d* = 0.74, *BF* = 3.18), but not significantly different between the 3-s and 5-s conditions (*t*[12] = 0.85, *p* = 0.82, *d* = 0.24, *BF* = 1.98), between the 5-s and 7-s conditions (*t*[12] = 2.33, *p* = 0.11, *d* = 0.65, *BF* = 0.38) or between the 7-s and 9-s conditions (*t*[12] = 0.43, *p* = 0.82, *d* = 0.12, *BF* = 0.30). These findings indicate that, in the continuous action condition, the reproduction of the encoded duration was prolonged (i.e., the subjective time regarding the reproduction tone was compressed) when the durations were 3 s and 5 s, but this subjective time compression decreased with increasing duration lengths and became accurate when the durations were 7 s and 9 s. In addition, in the single action condition, the encoded duration was accurately reproduced (i.e., the subjective time regarding the reproduction tone was accurate), regardless of the duration.

Figure 2B shows the regression lines fitted to each condition’s reproduction time error and steering wheel velocity in the continuous action condition: (the reproduction time error) = w0 + w1 × (the steering wheel velocity). The slopes of the lines appeared to be positive. A one-sample t-test indicated that the correlation coefficients between these metrics, as shown in Fig. 2C, were significantly higher than 0 (*t*[3] = 3.38, *p* = 0.043, *d* = 1.69, *BF* = 2.63). This finding indicates a positive relationship between subjective time compression and the speed of action.

To summarize, in Experiment 1, we found that the continuous action but not the single action compressed subjective time regarding the reproduction tone, which was presented as feedback for the action, particularly when the durations were 3 s and 5 s, and that the subjective time compression induced by the continuous action decreased with increasing duration lengths, becoming accurate when the durations were 7 s and 9 s. Furthermore, the subjective time compression induced by the continuous action was correlated with the speed of continuous action.

### Experiment 2

**Figure 3:**
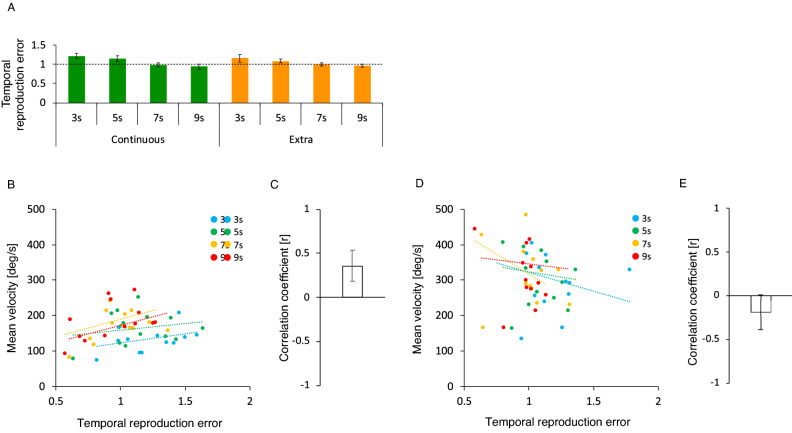
Average temporal reproduction error for the continuous and extra continuous action conditions (**A**), regression lines fitted to each duration condition’s temporal reproduction error, and steering wheel velocity for the continuous action condition (**B**) and for the extra continuous action conditions (**D**), and its average correlation coefficient for the continuous action condition (**C**) and for the extra continuous action conditions (**E**). Statistical comparisons revealed that, compared with 1 (a dotted line), the temporal reproduction errors in both conditions were higher in the 3-s and 5-s conditions, but identical in the 7-s and 9-s conditions (**A**). The slopes of the regression lines appeared to be positive in the continuous action condition (**B**) and negative in the extra continuous action condition (**D**), and the average correlation coefficient was significantly higher than 0 in the continuous action condition (**C**) but marginally lower than 0 in the extra continuous action condition. Error bars represent 95% confidence intervals.

Figure 3A shows the average temporal reproduction error for the continuous and extra continuous action conditions. Both conditions appeared to show similar trends (i.e., the temporal reproduction errors were higher than a value of 1 in the 3-s and 5-s conditions, but lower than a value of 1 in the 7-s and 9-s conditions). A one-sample *t*-tests revealed that, compared with a value of 1, temporal reproduction errors in the continuous action condition were significantly higher in the 3-s condition (*t*[12] = 3.23, *p* = 0.0072, *d* = 0.89, *BF* = 7.54), marginally higher in the 5-s condition (*t*[12] = 2.02, *p* = 0.066, *d* = 0.56, *BF* = 1.30), but not significantly different in the 7-s and 9-s conditions (*t*[12] = 0.25, *p* = 0.806, *d* = 0.07, *BF* = 0.29, *t*[12] = 0.89, *p* = 0.389, *d* = 0.25, *BF* = 0.39, respectively), and the temporal reproduction errors in the extra continuous action condition were significantly higher in the 3-s condition (*t*[12] = 2.23, *p* = 0.042, *d* = 0.63, *BF* = 1.83), marginally higher in the 5-s condition (*t*[12] = 1.85, *p* = 0.088, *d* = 0.51, *BF* = 1.10), but not significantly different in the 7-s and 9-s conditions (*t*[12] = 0.03, *p* = 0.973, *d* = 0.01, *BF* = 0.28, *t*[12] = 1.01, *p* = 0.329, *d* = 0.28, *BF* = 0.43, respectively). In addition, temporal reproduction errors were not significantly different between the continuous and extra continuous action conditions in Fig. 3A. As expected, a two-way repeated measures ANOVA showed no main effect of task condition (*F*[1, 12] = 0.26, *p* = 0.619, *ηp*^2^ = 0.02, *BF* = 0.20), a significant main effect of duration condition (*F*[3, 36] = 16.16, *p* < 0.001, *ηp*^2^ = 0.57, *BF* = 38,649.53) and no interaction between task and duration condition (*F*[3, 36] = 0.59, *p* = 0.625, *ηp*^2^ = 0.05, *BF* = 0.14). Post-hoc tests for the main effect of duration condition revealed that the temporal reproduction error was significantly larger in the 3-s condition compared with the 7-s and 9-s conditions (*t*[12] = 4.46, *p* = 0.002, *d* = 1.24, *BF* = 162.22, *t*[12] = 4.49, *p* = 0.002, *d* = 1.24, *BF* = 744.35, respectively), significantly larger in the 5-s condition than the 7-s and 9-s conditions (*t*[12] = 5.15, *p* = 0.001, *d* = 1.43, *BF* = 112.34, *t*[12] = 5.30, *p* = 0.001, *d* = 1.47, *BF* = 91.77, respectively), but not significantly different between the 3-s and 5-s conditions (*t*[12] = 1.51, *p* = 0.185, *d* = 0.42, *BF* = 0.70) and between the 7-s and 9-s conditions (*t*[12] = 1.82, *p* = 0.185, *d* = 0.51, *BF* = 0.54). These findings suggest that, in both task conditions, the reproduced encoded duration was prolonged (i.e., the subjective time regarding the reproduction tone was compressed) when the durations were 3-s and 5-s, but this subjective time compression decreased with increasing duration length, becoming accurate when the durations were 7-s and 9-s.

Figure 3B, D show the regression lines fitted to each condition’s reproduction time error and steering wheel velocity (the continuous action condition: B, the extra continuous action condition: D). The slopes of the lines were positive in the continuous action condition and negative in the extra continuous action condition. A one-sample t-test revealed that the correlation coefficients between these metrics were significantly higher than 0 in the continuous action condition (*t*[3] = 6.39, *p* = 0.007, *d* = 3.20, *BF* = 8.34), as shown in Fig. 3C, but were marginally lower than 0 in the extra continuous action condition (*t*[3] =  − 2.98, *p* = 0.059, *d* = 1.49, *BF* = 2.13], shown in Fig. 3E. These findings indicate a positive relationship between the subjective time compression and the speed of action in the continuous action condition but a weak negative relationship in the extra continuous action condition.

To summarize, in Experiment 2, in the continuous action condition, we replicated the trends found in Experiment 1. Furthermore, we found that even the extra continuous action compressed the subjective time regarding the reproduction tone which is presented as feedback for the combination of single actions (i.e., two key presses), particularly when the durations were 3 s and 5 s, and that the subjective time compression induced by the extra continuous action decreased with increasing duration lengths, becoming accurate when the durations were 7 s and 9 s. However, unlike the continuous action condition, in the extra continuous action condition, the subjective time compression induced by the extra continuous action was negatively correlated with the speed of continuous action.

## Discussion

There is increasing evidence that voluntary actions can modulate the subjective time experience of its outcomes, regardless of the sensory modality, optimizing dynamic interaction with the external environment. However, no previous study has examined whether different types of voluntary actions modulate the subjective time experience of action outcomes differently. Elucidating the effects of different voluntary action types on the subjective time experience of action outcomes may help people to modulate their own experience of the duration of daily events intentionally through their voluntary actions.

In the present study, using a temporal reproduction task in which participants reproduce the duration of an auditory stimulus to which they were previously exposed by performing different types of voluntary action with a common auditory feedback, we examined how the subjective time experience of action outcomes changed with voluntary action types. We contrasted a combination of single actions and a continuous action in Experiment 1 and contrasted task-related continuous action and task-unrelated continuous action in Experiment 2. In Experiment 1, we found that the continuous action but not the combination of single actions compressed the subjective time regarding the reproduction tone, which was presented as the action outcome, where the subjective time compression induced by the continuous action was positively correlated with the speed of continuous action. The subjective time compression induced by the continuous action cannot be explained by an increase in physical workload, which has been reported to influence perception in other domains. For example, perceived distance increases and hills seem steeper under conditions requiring greater physical workload (e.g., when carrying a heavy load)^38–40^. In the current study, the physical workload would be expected to be larger for the continuous action compared with the single action, and would be expected to increase with the increase of the speed of the continuous action. However, a meta-analysis investigating the effect of physical workload on duration judgements revealed that increasing physical workload results in longer perceived duration of various tasks^41^. This direction of the effect of physical workload appears to be opposite to the pattern observed in our current findings. In addition, the elongation of subjective time as a result of workload has also been found for mental activity. A meta-analysis showed that depleted attentional resources and increased cognitive load result in longer retrospective subjective time judgements^42^. This direction of the effect of mental workload also appears to be opposite to the pattern observed in our current findings because mental workload, such as the build-up of fatigue, would be expected to be large during the continuous action and to increase with an increase in the speed of the continuous action. Thus, it is plausible that continuity of action compressed the subjective time experience of the action outcome (i.e., the reproduction tone) to maintain the experience of controlling the action outcome, as has been repeatedly demonstrated in previous studies^12–16^, and the experience of controlling action outcomes in the external environment is referred to as the sense of agency^43,44^. The current findings are novel because the majority of previous studies have focused on the subjective time experience of action outcomes when actions are performed voluntarily or not^12–20^ and no previous study has shown that continuity of action plays a role in maintaining the sense of agency.

The conclusion that continuity of action plays a role in maintaining the sense of agency seems to be in accord with the internal comparator model^45,46^, which is a theoretical framework regarding the generation of the sense of agency supported by a substantial number of studies using a variety of paradigms and indices^47–53^. According to this model, the sense of agency is produced via a comparison between the predicted outcome of an action which is generated by means of an internal forward model^54,55^, and its actual outcome. If predicted and actual outcomes match, people are more likely to feel that the perceived event occurred through their actions, leading them to experience a sense of agency. In contrast, if there is a mismatch between predicted and actual outcomes, people typically experience a weaker sense of agency. In the current study, performing a continuous action (i.e., continuously turning the steering wheel) provided more opportunities to create matches between the predicted and the actual outcomes compared with performing the single action (i.e., two key presses), because, while performing the continuous action, participants could predict the action outcomes, compare them with the actual outcomes and accumulate the matches more than twice, which was the maximum number of opportunities to predict the action outcomes when performing the single action (i.e., at the beginning and end of the reproduction tone by pressing the key). The increased number of matches in performing the continuous action would be expected to contribute to maintaining (or enhancing) the sense of agency to a greater extent. The finding that the subjective time compression induced by the continuous action was positively correlated with the speed of the action can be also explained in this way. That is, it is predictable that the number of matches was higher for the faster continuous action than the slower continuous action, because, in the faster continuous action condition, the number of actions was larger, and thus the number of opportunities for predicting the action outcomes during continuous action was greater compared with the slower continuous action condition.

Interestingly, even the task-unrelated continuous action (i.e., the extra continuous action) compressed the subjective time regarding the reproduction tone which was presented as the action outcome of the combination of single actions in Experiment 2. However, unlike the task-related continuous action, the subjective time compression induced by the task-unrelated continuous action was negatively correlated with the speed of continuous action, suggesting that the mechanism underlying the subjective time compression induced by the task-unrelated continuous action may differ from the mechanism of subjective time compression induced by the task-related continuous action. This subjective time compression induced by the task-unrelated continuous action appears to be explained by a lack of attention to the reproduction tone, which was presented as the action outcome of the combination of single actions. It has been reported that, when the lack of attention to an event is induced by dividing attentional resources, the time experience of the event’s duration is subjectively compressed^56^. In the case of the task-unrelated continuous action, performing the extra continuous action could divide participants’ attentional resources between performing the temporal reproduction task via the combination of single actions with the right hand and performing an extra continuous action with the left hand. The current finding of a negative relationship between the subjective time compression induced by the task-unrelated continuous action and the speed of the action can also be explained in this way. Thus, it is predictable that the increased speed of the extra continuous action with the left hand consumed more attentional resources, resulting in a lack of attention to the reproduction tone which was presented as the action outcome of the combination of single actions with the right hand, inducing the subjective time compression of the reproduction tone.

The subjective time compression induced by both task-related and task-unrelated continuous actions was found to decrease with increasing duration lengths through Experiments 1 and 2. This trend, which is partially in agreement with Vierordt’s law^57^ (i.e., when multiple intervals are presented within the same block, long intervals are under-reproduced and short intervals are over-reproduced), appears to be a robust replication of the findings of previous studies employing the temporal reproduction task^58–62^. In accord with these previous studies, with increasing duration lengths, the reproduced durations were progressively under-reproduced relative to physical time. This trend also appears to be modulated by different reproduction methods (for example, maintaining a keypress throughout the duration results in a stronger trend than pressing the key to start and stop the duration)^63^ and by the individual’s reaction times, because preparing and executing motor responses for reproduction takes time^64^. In the current experiments, the subjective time compression induced by both task-related and task-unrelated continuous actions seemed to conflict with those task-relevant factors. If so, the temporal reproduction task may have limitations for investigating the subjective time experience of action outcomes.

In addition to this methodological limitation, the present study involved several other limitations that should be considered. First, because we employed durations of 3 s, 5 s, 7 s and 9 s for reproduction in the experimental task, our findings are limited to perception of stimuli above the 1-s time scale. A large body of neuroimaging studies have shown evidence of distinct neural systems for measuring below the 1-s and above the 1-s durations^1,2,4,65,66^. The neural system, which mainly comprises subcortical structures, is likely to play a role in the estimation of time durations below the 1-s range, and another neural system, which mainly comprises cortical areas, is thought to be more related to the estimation of time durations above the 1-s range. Therefore, future studies should investigate whether the current findings regarding the subjective time compression induced by both task-related and task-unrelated continuous actions are generalized to perception of stimuli below the 1-s time scale. Second, in the current study, we did not control for the speed of turning the steering wheel, and participants turned it at a self-selected comfortable constant speed. Therefore, our findings regarding the relationship between the speed of continuous action and subjective time compression could not be completely separated from individual differences. It has been reported that some individual differences can influence performance in temporal reproduction tasks. For example, impulsive individuals tend to reproduce shorter time durations for stimuli above 1 s in duration, compared with individuals who are less impulsive^67,68^. To clarify the relationship between the speed of continuous action and subjective time compression, the speed of continuous action should be controlled as a within-subjects factor. Future well-controlled studies that replicate the current findings may be valuable for gaining a deeper understanding of subjective time compression induced by both task-related and task-unrelated continuous actions. Third, in the present study, there were borderline cases where the *p*-values showed significance but the BFs did not quite reach the positive evidence value (i.e., 3 > *BF* > 1), or, in contrast, where the *p*-values did not show significance but the BFs provided weak support for the null hypothesis (i.e., 1 > *BF* > 0.33). In the borderline cases, the evidence based on the *p*-values could be statistically weakened to some extent. Some of the borderline cases may be indirectly supported by some other results which have reached the positive evidence value. For example, a borderline case of a one-sample t-test to compare the mean correlation coefficients with 0 in the continuous action condition in Experiment 1 (*p* = 0.043, *BF* = 2.63) may be indirectly supported by being replicated with the positive evidence value in Experiment 2 (*p* = 0.007, *BF* = 8.34). However, not every borderline case can be supported in this way. It has been suggested that obtaining more data will be important to strengthen the evidence in borderline cases^37^. The current sample size might be not enough to detect real effects found in the present study, which could restrict the generalizability of the current findings. Replication by studies with a larger sample size are needed to strengthen the current findings.

An interesting possibility regarding the application of our findings is related to developing effective methods for helping people to intentionally modulate their own time experience of daily events through their voluntary actions. The current findings suggest two potential methods to achieve this. First, adding an extra action to a main action task could be useful for dividing attentional resources. However, this approach may not be practical because it has been reported that divided attention can cause a decline in the performance of a manual task. For example, divided attention induced by performing a secondary task is reported to contribute to instability of balance control during standing and walking in both healthy and older adults (for review, see Woollacott and Shumway-Cook^69^). Another possible approach is to enhance the sense of agency over the task-related action. The current results suggested that increasing the number of matches between the predicted and actual action outcomes of a task-related action may contribute to compression of the subjective time experience of the action outcome, enhancing the sense of agency. Investigating effective methods for compressing the subjective time experience by enhancing the sense of agency is a potentially interesting direction for future research. Providing additional types of sensory feedback could potentially increase the number of matches between the predicted and the actual action outcomes, instead of increasing the speed of the action. This possibility could be tested by examining changes in subjective time compression associated with changes in the amount of sensory feedback for the task-related action.

## Conclusions

The current results revealed that the subjective time experience of action outcomes was compressed by continuous action, and that this effect could not be explained by increased physical workload. This finding suggests that subjective time compression induced by continuous action plays a role in enhancing the experience of controlling the action outcome, as has been repeatedly shown in previous studies. In addition, we found that the subjective time experience of action outcomes was also compressed if the action included an extra task-unrelated continuous action, which appeared to be explained by the lack of attention to action outcomes. These findings are novel because the majority of previous studies have focused on the subjective time experience of action outcomes when actions were performed voluntarily or not, and no previous study has focused on the effect of differences in voluntary action types on the subjective time experience of action outcomes. Our findings may be useful for developing methods by which people can intentionally compress their own time experience of daily events via their voluntary actions. Further work will be required to establish the generalizability of the current findings to practical settings.

## Data Availability

The data that support the findings of this study are available from the corresponding author upon reasonable request.

## References

[1] Merchant H, Yarrow K (2016). How the motor system both encodes and influences our sense of time. Curr. Opin. Behav. Sci..

[2] Lewis PA, Miall RC (2003). Distinct systems for automatic and cognitively controlled time measurement: evidence from neuroimaging. Curr. Opin. Neurobiol..

[3] Wiener M, Turkeltaub P, Coslett HB (2010). The image of time: a voxel-wise meta-analysis. Neuroimage.

[4] Radua, J., Pozo, N. O., Goḿez, J., Guillen-Grima, F. & Ortuño, F. Meta-analysis of functional neuroimaging studies indicates that an increase of cognitive difficulty during executive tasks engages brain regions associated with time perception. *Neuropsychologia* **58**, 14–22 (2014). 10.1016/j.neuropsychologia.2014.03.016 24709569

[5] Nani A, Manuello J, Liloia D, Duca S, Costa T, Cauda F (2019). The neural correlates of time: a meta-analysis of neuroimaging studies. Cognit. Neurosci..

[6] Teghil A, Boccia M, D'Antonio F, Di Vita A, de Lena C, Guariglia C (2019). Neural substrates of internally-based and externally-cued timing: an activation likelihood estimation (ALE) meta-analysis of fMRI studies. Neurosci. Biobehav. Rev..

[7] Cona, G., Wiener, M. & Scarpazza, C. From ATOM to GradiATOM: Cortical gradients support time and space processing as revealed by a meta-analysis of neuroimaging studies. *bioRxiv.* (2020). 10.1016/j.neuroimage.2020.117407 32992001

[8] Pastor MA, Artieda J, Jahanshahi M, Obeso JA (1992). Time estimation and reproduction is abnormal in parkinson’s disease. Brain.

[9] Avanzino L (2009). Cerebellar involvement in timing accuracy of rhythmic finger movements in essential tremor. Eur. J. Neurosci..

[10] Lucas M (2013). Time perception impairs sensory-motor integration in Parkinson’s disease. Int. Arch. Med..

[11] Pedrosa DJ (2016). Time reproduction deficits in essential tremor patients. Mov. Disord..

[12] Haggard P, Clark S, Kalogeras J (2002). Voluntary action and conscious awareness. Nat. Neurosci..

[13] Engbert K, Wohlschläger A, Thomas R, Haggard P (2007). Agency, subjective time, and other minds. J. Exp. Psychol. Hum. Percept. Perform..

[14] Engbert K, Wohlschläger A, Haggard P (2008). Who is causing what? The sense of agency is relational and efferent-triggered. Cognition.

[15] Humphreys GR, Buehner MJ (2009). Magnitude estimation reveals temporal binding at super-second intervals. J. Exp. Psychol. Hum. Percept. Perform..

[16] Imaizumi, S., Tanno, Y. & Imamizu, H. Compress global, dilate local: Intentional binding in action–outcome alternations. *Conscious Cognit.* **73**, 102768. (2019). 10.1016/j.concog.2019.102768 31254736

[17] Yarrow K, Haggard P, Heal R, Brown P, Rothwell JC (2001). Illusory perceptions of space and time preserve cross-saccadic perceptual continuity. Nature.

[18] Park J, Schlag-Rey M, Schlag J (2003). Voluntary action expands perceived duration of its sensory consequence. Exp. Brain Res..

[19] Yarrow K, Rothwell JC (2003). Manual chronostasis: tactile perception precedes physical contact. Curr Biol..

[20] Imaizumi S, Asai T (2017). My action lasts longer: potential link between subjective time and agency during voluntary action. Conscious Cognit..

[21] Faul F, Erdfelder E, Lang AG, Buchner A (2007). G* Power 3: a flexible statistical power analysis program for the social, behavioral, and biomedical sciences. Behav. Res. Methods.

[22] https://www.researchgate.net/post/How_compute_a_repeated_measure_power_analysis_in_Gpower

[23] Shirai, R., & Ogawa, H. Affective evaluation of images influences personality judgments through gaze perception. *PloS one* **15**, e0241351 (2020). 10.1371/journal.pone.0241351 PMC7643958 33151950

[24] Oldfield RC (1971). The assessment and analysis of handedness: the Edinburgh inventory. Neuropsychologia.

[25] Itkonen M, Costa Á, Yamasaki H, Okajima S, Alnajjar F, Kumada T, Shimoda S (2019). Influence of bimanual exercise on muscle activation in post-stroke patients. ROBOMECH J.

[26] Brainard DH (1997). The psychophysics toolbox. Spat. Vis..

[27] Brown SW (1985). Time perception and attention: the effects of prospective versus retrospective paradigms and task demands on perceived duration. Percept. Psychophys..

[28] Brown SW (1997). Attentional resources in timing: Interference effects in concurrent temporal and nontemporal working memory tasks. Percept. Psychophys..

[29] Glicksohn J, Hadad Y (2012). Sex differences in time production revisited. J. Individ. Dif..

[30] Creelman CD (1962). Human discrimination of auditory duration. J. Acoust. Soc. Am..

[31] Treisman M (1963). Temporal discrimination and the indifference interval: implications for a model of the ‘internal clock’. Psychol. Monogr..

[32] Church RM (1984). Properties of the internal clock. Ann. N. Y. Acad. Sci..

[33] Gibbon J, Malapani C, Dale CL, Gallistel CR (1997). Toward a neurobiology of temporal cognition: advances and challenges. Curr. Opin. Neurobiol..

[34] Shaffer JP (1986). Modified sequentially rejective multiple test procedures. J. Am. Stat. Assn..

[35] Westfall PH, Johnson WO, Utts JM (1997). A Bayesian perspective on the Bonferroni adjustment. Biometrika.

[36] Goss-Sampson, M. A. *Bayesian Inference in JASP: A Guide for Students.* http://static.jasp-stats.org/Manuals/Bayesian_Guide_v0_ 12_2_1.pdf (2020).

[37] Keysers C, Gazzola V, Wagenmakers EJ (2020). Using Bayes factor hypothesis testing in neuroscience to establish evidence of absence. Nat. Neurosci..

[38] Bhalla M, Proffitt DR (1999). Visual-motor recalibration in geographical slant perception. J. Exp. Psychol. Hum. Percept. Perform..

[39] Sugovic M, Witt JK (2013). An older view on distance perception: older adults perceive walkable extents as farther. Exp. Brain Res..

[40] Witt JK, Proffitt DR, Epstein W (2004). Perceiving distance: a role of effort and intent. Perception.

[41] Block RA, Hancock PA, Zakay D (2016). Physical load affects duration judgements: a meta-analytic review. Acta Psychol..

[42] Block RA, Hancock PA, Zakay D (2010). How cognitive load affects duration judgments: a meta-analytic review. Acta Psychol..

[43] Haggard P (2017). Sense of agency in the human brain. Nat. Rev. Neurosci..

[44] Haggard P, Chambon V (2012). Sense of agency. Curr. Biol..

[45] Blakemore S-J, Frith CD, Wolpert DM (1999). Spatio-temporal prediction modulates the perception of self-produced stimuli. J. Cognit. Neurosci..

[46] Frith C, Blakemore S-J, Wolpert DM (2000). Explaining the symptoms of schizophrenia: abnormalities in the awareness of action. Brain Res. Rev..

[47] Ebert JP, Wegner DM (2010). Time warp: authorship shapes the perceived timing of actions and events. Conscious Cognit..

[48] Farrer C, Valentin G, Hupé JM (2013). The time windows of the sense of agency. Conscious Cognit..

[49] Hon N, Poh J-H, Soon C-S (2013). Preoccupied minds feel less control: Sense of agency is modulated by cognitive load. Conscious Cognit..

[50] Kawabe T (2013). Inferring sense of agency from the quantitative aspect of action outcome. Conscious Cognit..

[51] Kühn, S., Nenchev, I., Haggard, P., Brass, M., Gallinat, J. & Voss, M. Whodunnit? Electrophysiological correlates of agency judgements. *PLoS ONE* **6**, e28657 (2011). 10.1371/journal.pone.0028657 PMC3237473 22194878

[52] Sato A, Yasuda A (2005). Illusion of sense of self-agency: Discrepancy between the predicted and actual sensory consequences of actions modulates the sense of self-agency, but not the sense of self-ownership. Cognition.

[53] Wen W, Yamashita A, Asama H (2015). The influence of action-outcome delay and arousal on sense of agency and the intentional binding effect. Conscious Cognit..

[54] Wolpert DM, Ghahramani Z, Flanagan JR (2001). Perspectives and problems in motor learning. Trends Cognit. Sci..

[55] Wolpert DM, Ghahramani Z, Jordan MI (1995). An internal model for sensorimotor integration. Science.

[56] Coull JT, Vidal F, Nazarian B, Macar F (2004). Functional anatomy of the attentional modulation of time estimation. Science.

[57] Vierordt, K. *Der Zeitsinn nach Versuchen.* (Laupp, Tubingen, Germany, 1868).

[58] Eisler, H. & Eisler, A, D. Time perception: effects of sex and sound intensity on scales of subjective duration. *Scand. J. Psychol. 33*, 339–358 (1992). 10.1111/j.1467-9450.1992.tb00923.x 1287826

[59] Noulhiane M, Mella N, Samson S, Ragot R, Pouthas V (2007). How emotional auditory stimuli modulate time perception. Emotion.

[60] Sawyer TF, Meyers PJ, Huser SJ (1994). Contrasting task demands alter the perceived duration of brief time intervals. Percept. Psychophys..

[61] Ulbrich P, Churan J, Fink M, Wittmann M (2007). Temporal reproduction: further evidence for two processes. Acta Psychol..

[62] Wittmann M, Simmons AN, Aron JL, Paulus MP (2010). Accumulation of neural activity in the posterior insula encodes the passage of time. Neuropsychologia.

[63] Mioni G, Stablum F, McClintock SM, Grondin S (2014). Different methods for reproducing time, different results. Atten. Percept. Psychophys..

[64] Droit-Volet S (2010). Stop using time reproduction tasks in a comparative perspective without further analyses of the role of the motor response: The example of children. Eur. J. Cognit. Psychol..

[65] Ortuño F, Guillen-Grima F, Lopez-Garcia P, Gomez J, Pla J (2011). Functional neural networks of time perception: Challenge and opportunity for schizophrenia research. Schizophr. Res..

[66] Schwartze M, Rothermich K, Kotz SA (2012). Functional dissociation of pre-SMA and SMA-proper in temporal processing. Neuroimage.

[67] Wittmann M, Paulus MP (2008). Decisionmaking, impulsivity and time perception. Trends Cognit. Sci..

[68] Wittmann M, Simmons AN, Flagan T, Lane SD, Wackermann J, Paulus MP (2011). Neural substrates oftime perception and impulsivity. Brain Res..

[69] Woollacott M, Shumway-Cook A (2002). Attention and the control of posture and gait: a review of an emerging area of research. Gait Posture..

